# Bacterial contamination of Pakistani currency notes from hospital and community sources

**DOI:** 10.12669/pjms.345.15477

**Published:** 2018

**Authors:** Hasan Ejaz, Azra Javeed, Muhammad Zubair

**Affiliations:** 1 Hasan Ejaz, PhD, Department of Clinical Laboratory Sciences, College of Applied Medical Sciences, Jouf University, Saudi Arabia; 2 Azra Javeed, M.Phil Scholar, Department of Microbiology, The Children’s Hospital and The Institute of Child Health, Ferozepur Road, Lahore, Pakistan; 3 Muhammad Zubair, M.Phil, Department of Microbiology, The Children’s Hospital and The Institute of Child Health, Ferozepur Road, Lahore, Pakistan

**Keywords:** Bacteria, Banknotes, Contamination, Pakistani currency

## Abstract

**Objective:**

We determined the bacterial contamination and antibiotic resistance profile of circulating Pakistani currency notes collected from hospital and community sources.

**Methods:**

This prospective study was organized from July to December 2015 in the Microbiology Department of The Children’s Hospital and The Institute of Child Health Lahore. It was done on one hundred currency notes of four different denominations collected from various groups of people in sterile polythene bags. Gram staining, colony morphology and various biochemical tests were used to identify the bacterial isolates. Kirby Bauer disc diffusion method was used to observe the antibacterial drug resistance.

**Results:**

There were 11 different types of bacterial species which contaminated 97 (97%) currency notes. The bacterial isolates discovered from paper currency notes included *Klebsiella* spp. (26.0%), Coagulase-negative *Staphylococci* (CoNS) (18.3%), *E. coli* (14.5%), *Pseudomonas* spp. (13.7%), *Citrobacter* spp. (11.5%), *Enterobacter* spp. (5.3%), *Acinetobacter* spp. (5.3%), *Streptococcus* spp. (2.3%), *Shigella* spp. (1.5%), *Salmonella* spp. (0.8%) and *Pantoea* spp. (0.8%). Most of the Gram-positive isolates were resistant to penicillin and ampicillin. None of the Gram-negative isolates found to be resistant to amikacin, cefoperazone-sulbactam and piperacillin-tazobactam.

**Conclusion:**

The currency notes circulating in hospital and community are contaminated with highly pathogenic and some multi-drug resistant bacteria. These currency notes could be a potential source of nosocomial and community-acquired infections.

## INTRODUCTION

Microorganisms are present almost everywhere in our surroundings. They may propagate via food, water, air and most importantly by fomites.^1^ Microbiological contamination refers to an accidental or non-intended introduction of infectious material like bacteria, virus, fungi, yeast, mould, protozoa or their toxins and by-products.^2^

Currency in the form of paper notes and coins, issued by the government and circulated within an economy used as a medium of exchange for goods and services.^3^ There are several sources of contamination of currency notes which could be from production, handling, usage, storage or atmosphere.^4^ Currency notes can be contaminated by contaminated hands, sneezing, coughing droplets or by tongue-wet fingers during money counting.^5^ *Klebsiella* spp. *Staphylococcus aureus, E.coli, Proteus* spp. *Salmonella* spp. *Pseudomonas* spp. *Bacillus* spp. Coagulase-negative *Staphylococci*, and *Micrococcus* spp. are among the organisms isolated from the currency notes.^6^ The highest number of the presence of *Escherichia coli* and Coagulase negative *Staphylococci* have been reported in currency notes.^7^ Acid-fast bacilli are also isolated from currency notes in circulation.^8^

Currency notes of lower denominations exhibit higher contamination level concerning currency notes of higher denominations due to their more rapid turnover.^9^ Bacterial contamination also depends upon the age of currency and the material used in the production of the currency note.^10^

Inanimate objects like currency play a significant role in the indirect transmission of infections like trachoma, diphtheria, gastroenteritis, whooping cough and diarrhoea.^11^ The contaminated money has a significant role in the transmission of multidrug-resistant microorganisms such as Methicillin-Resistant *Staphylococcus aureus* (MRSA) and Vancomycin-resistant *Enterococci* (VRE). The multidrug-resistant nosocomial pathogen may survive for an extended period on the hospital surfaces and could be transmitted to others through currency notes.^12^ Only a few studies from Pakistan highlighted the significance of microbial contamination of currency notes in the community but without notifying the possible transmission of multi-drug resistant pathogens in hospitals. We aimed this study to report the bacterial contamination and antibiotic resistance profile of circulating Pakistani currency notes collected from hospital and community sources.

## METHODS

This prospective study was organized from July to December 2015 in the Microbiology Department of The Children’s Hospital and The Institute of Child Health Lahore, Pakistan after the approval by the institutional ethical committee under Reference no. 03/140/16. A total number of hundred currency notes of various denominations were collected from hospital staff, butcher’s shops, vegetable vendors and public transport conductors in exchange for higher denomination currency notes. We collected denominations of 10, 20, 50 and 100 rupees circulation among the various groups of people. The currency notes were obtained by wearing sterile latex gloves and placed immediately into sterile polythene envelopes. Samples were labeled and transported directly to the lab for microbiological analysis.

We submerged currency notes in Brain Heart Infusion Broth (BHI) and incubated overnight at 37°C. The broth cultures were sub-cultured on Blood, Chocolate and MacConkey agar plates. The culture plates were incubated aerobically at 37°C for another 24 hours. We use Lowenstein-Jensen egg-based media for the possible isolation of Mycobacteria. The samples cultured on Lowenstein-Jensen incubated at 37°C for up to eight weeks. The mixed cultures on Blood, Chocolate and MacConkeyagar plates containing the growth of more than one bacteria were further sub-cultured on Blood and MacConkey agar to isolate each bacteria. The purified single well-isolated colonies were identified by Gram’s staining, ZiehlNeelsen staining, biochemical reaction and API 20-E (BioMerieux, France).

The antimicrobial drug resistance was performed using modified Kirby-Bauer disc diffusion technique. The bacterial colonies suspended in sterile saline and turbidity adjusted according to the McFarland 0.5 turbidity standards. A sterile swab was used to streak the suspension on the Mueller Hinton agar plates. Mueller Hinton agar plates incubated overnight at 37°C. Drug resistance was observed by using two separate panels of antibiotics against each of the Gram-positive and Gram-negative bacteria. We used amikacin, co-amoxiclav, cefotaxime, ceftriaxone, ceftazidime, cefuroxime, cefixime, ciprofloxacin, imipenem, cefoperazone-sulbactam, piperacillin-tazobactam and meropenem antibiotic discs for Gram-negative bacteria. Amikacin, gentamicin, co-amoxiclav, ampicillin, oxacillin, ciprofloxacin, vancomycin, linezolid, cefotaxime, ceftriaxone, cefuroxime, cefixime, ceftazidime and penicillin antibiotic discs were used against for Gram-positive bacteria. Methicillin resistance was detected by using cefoxitin disc and ESBL production was reported on the basis of double disc synergy test. The zones of inhibition measured in mm and interpreted according to the Clinical and Laboratory Standards Institute (CLSI) guidelines.^13^ The data was analysed using IBM SPSS version 23.0 (Statistical Package for Social Sciences).

## RESULTS

Out of the total number of 100 currency notes, 97 (97%) were contaminated with 11 different species of Gram-positive and Gram-negative bacteria. Only three (3%) currency notes collected from the hospital Pharmacy showed no growth of bacteria. A total number of 131 bacterial strains isolated from 97 contaminated notes which included 104 (79.4%) Gram-negative and 27 (20.6%) Gram-positive bacteria. There were 40 (40%) notes which exhibited growth of more than one bacterial strain. We isolated 34 (26.0%) *Klebsiella* spp., 24 (18.3%) Coagulase-negative *Staphylococcus*, 19 (14.5%) *E. coli*, 18 (13.7%) *Pseudomonas* spp., 15 (11.5%) *Citrobacter* spp., 7(5.3%) *Enterobacter* spp., 7 (5.3%) *Acinetobacter* spp., (2.3%) *Streptococcus* spp. and (0.8%) *Pantoea* spp. None of the Acid Fast Bacilli (AFB) recovered from any of the specimens. There was a positive case of *Salmonella* spp. (0.8%) and *Shigella* spp. (0.8%) isolated from Butcher’s shop. The other case of *Shigella* spp. (0.8%) isolated from a currency note collected from a Ward boy (Table-I). Currency notes of lower denomination showed more contamination. Out of total 131 isolates, 46 were isolated from Rs. 10, 36 from Rs. 20, 30 from Rs. 50 and 19 bacterial strains were isolated from Rs. 100 (Table-II). Antimicrobial susceptibility testing revealed that none of the Gram-negative bacteria found to be resistant to amikacin, cefoperazone-sulbactam and piperacillin-tazobactam. Some of the isolates exhibited resistance to carbapenems, and other antibiotics have shown in the Table-III. The antibacterial drug resistance of the majority of the Gram-positive bacteria presented resistance to penicillin and ampicillin. Among the 24 species of CoNS, 8 (33.3%) species found to be resistant to oxacillin which was used as a screening drug for to detect methicillin resistance. Only 2 (7.4%) Gram-positive strains found to be resistant to linezolid and 1 (3.7%) to vancomycin. All the strains of *Streptococci* showed resistance to ampicillin, penicillin and cefuroxime (Table-IV).

**Table-I:** Distribution of bacteria among various sources.

Organism	Sources							Total n (%)

	Ward boys	Pharmacy	Patient’s attendants	Cafeteria	Butcher’s shops	Vendors	Public transport conductors	
*Klebsiella spp*.	4	2	7	3	4	9	5	34 (26.0)
Coagulase negative*Staphylococci* (CoNS)	6	0	7	2	4	2	3	24 (18.3)
*E.coli*	2	1	1	3	4	6	2	19 (14.5)
*Pseudomonas spp*.	1	1	3	0	5	3	5	18 (13.7)
*Citrobacter* spp.	0	1	4	1	2	5	2	15 (11.5)
*Enterobacter* spp.	1	0	2	1	1	1	1	7 (5.3)
*Acinetobacter* spp.	2	1	1	0	2	0	1	7 (5.3)
*Streptococcus* spp	0	0	1	0	2	0	0	3 (2.3)
*Shigella* spp.	1	0	0	0	1	0	0	2 (1.5)
*Salmonella* spp.	0	0	0	0	1	0	0	1 (0.8)
*Pantoea* spp.	0	0	0	1	0	0	0	1 (0.8)

Total	17 (13.0%)	6 (4.6%)	26 (19.8%)	11 (8.4%)	26 (19.8%)	26 (19.8%)	19 (14.5%)	131 (100%)

**Table II:** Denomination wise distribution of bacterial isolates

Organisms	Denomination				Total

	Rs. 10	Rs. 20	Rs. 50	Rs. 100	
*Klebsiella spp*.	12	10	7	5	34
*E. coli*	6	5	5	3	19
*Enterobacter spp*.	2	2	3	0	7
*Citrobacter spp*.	10	3	2	0	15
*Staphylococcus* (CoNS)	7	5	6	6	24
*Pseudomonas spp*.	7	8	2	1	18
*Acinetobacter spp*.	2	1	3	1	7
*Salmonella spp*.	0	0	0	1	1
*Shigella spp*.	0	1	0	1	2
*Pantoea spp*.	0	0	0	1	1
*Streptococcus spp*.	0	1	2	0	3

Total	46	36	30	19	131

**Table-III:** Antimicrobial resistance profile of Gram-negative bacteria (n=104).

Organism	Amikacin n (%)	Co-amoxiclav n (%)	Cefotaxime n (%)	Ceftriaxone n (%)	Ceftazidime n (%)	Cefuroxime n (%)	Cefixime n (%)	Ciprofloxacin n (%)	Imipenem n (%)	Cefoperazone-Sulbactam n (%)	Piperacillin-Tazobactam n (%)	Meropenem n (%)
*Klebsiella spp*. n=34	0 (0.0)	4 (11.8)	0 (0.0)	0 (0.0)	3 (8.8)	4 (11.8)	9 (26.5)	0 (0.0)	0 (0.0)	0 (0.0)	0 (0.0)	2 (5.9)
*E. coli* n=19	0 (0.0)	0 (0.0)	1 (5.3)	0 (0.0)	0 (0.0)	1 (5.3)	0 (0.0)	1 (5.3)	0 (0.0)	0 (0.0)	0 (0.0)	1 (5.3)
*Pseudomonas spp*. n=18	0 (0.0)	2 (11.1)	1 (5.6)	2 (11.1)	1 (5.6)	7 (38.9)	9 (50.0)	0 (0.0)	1 (5.6)	0 (0.0)	0 (0.0)	3 (16.7)
*Citrobacter spp*. n=15	0 (0.0)	1 (6.7)	0 (0.0)	2 (13.3)	0 (0.0)	0 (0.0)	5 (33.3)	0 (0.0)	0 (0.0)	0 (0.0)	0 (0.0)	2 (13.3)
*Enterobacter spp*. n=7	0 (0.0)	5 (71.4)	0 (0.0)	2 (28.6)	0 (0.0)	4 (57.1)	3 (42.9)	1 (14.3)	0 (0.0)	0 (0.0)	0 (0.0)	1 (14.3)
*Acinetobacter spp*. n=7	0 (0.0)	0 (0.0)	2 (28.6)	0 (0.0)	0 (0.0)	5 (71.4)	3 (42.9)	1 (14.3)	0 (0.0)	0 (0.0)	0 (0.0)	0 (0.0)
*Shigella spp*. n=2	0 (0.0)	0 (0.0)	0 (0.0)	0 (0.0)	0 (0.0)	0 (0.0)	0 (0.0)	0 (0.0)	0 (0.0)	0 (0.0)	0 (0.0)	0 (0.0)
*Salmonella spp*. n=1	0 (0.0)	0 (0.0)	0 (0.0)	0 (0.0)	0 (0.0)	0 (0.0)	0 (0.0)	0 (0.0)	0 (0.0)	0 (0.0)	0 (0.0)	0 (0.0)
*Pantoea spp*. n=1	0 (0.0)	0 (0.0)	0 (0.0)	0 (0.0)	0 (0.0)	0 (0.0)	0 (0.0)	0 (0.0)	0 (0.0)	0 (0.0)	0 (0.0)	0 (0.0)

Total	0 (0.0%)	12 (11.5%)	4 (3.8%)	6 (5.8%)	4 (3.8%)	21 (20.2%)	29 (27.9%)	3 (2.9%)	1 (1.0%)	0 (0.0%)	0 (0.0%)	9 (8.7%)

**Table-IV:** Antimicrobial resistance profile of Gram-positive bacteria (n=27)

Antibiotic	Coagulase negative Staphylococci (CoNS) n=24 n (%)	Streptococcus spp. n=3 n (%)	Total n (%)
Ampicillin	14 (58.3)	3 (100%)	17 (63.0)
Penicillin	14 (58.3)	3 (100%)	17 (63.0)
Cefuroxime	8 (33.3)	3 (100%)	11 (40.7)
Ceftriaxone	8 (33.3)	2 (66.7)	10 (37.0)
Cefixime	8 (33.3)	2 (66.7)	10 (37.0)
Ceftazidime	8 (33.3)	2 (66.7)	10 (37.0)
Co-amoxiclav	8 (33.3)	2 (66.7)	10 (37.0)
Cefotaxime	8 (33.3)	0 (0.0)	8 (29.6)
Ciprofloxacin	7 (29.2)	1 (33.3)	8 (29.6)
Oxacillin	8 (33.3)	NA	8 (33.3)
Amikacin	0 (0.0)	2 (66.7)	2 (7.4)
Gentamicin	0 (0.0)	2 (66.7)	2 (7.4)
Linezolid	2 (8.3)	0 (0.0)	2 (7.4)
Vancomycin	1 (4.2)	0 (0.0)	1 (3.7)

**NA:** Not Applied

## DISCUSSION

Our study revealed the occurrence of 97% bacterial contamination from Pakistani currency notes which could be a potential source of infectious diseases in healthy as well as immunocompromised patients. A contamination rate of 92.5% reported by another study from Lahore, Pakistan which is a little lower than our results.^14^ A study from Nigeria reported 52.5% contamination and another study from Saudi Arabia observed 92% contaminants in the currency notes.^7^,^15^ We found a higher rate of currency contamination which could be due to the inclusion of samples from the hospital sources which contains a large number of nosocomial pathogens. Some studies on Ghanaian and Indian currency reported a contamination rate of 98.6% and 93.9%, respectively which is close to the results of our study.^16^,^17^

We observed 26.0% *Klebsiella* spp., 18.3% Coagulase-negative *Staphylococcus*, 14.5% *E. coli* and 13.7% *Pseudomonas* spp. while rest of the organisms were less in number. A study on Nigerian currency revealed the higher occurrence of *Staphylococcus* spp.^18^ An Indian study reported the presence of *Enterococcus* spp. as the most common pathogen.^5^ A survey conducted on the Bangladeshi currency notes (Taka) found a higher rate of multi-drug resistant bacterial contaminants of *Salmonella* and *Shigella* spp. which were mostly isolated from fish and poultry sellers.^19^ We observed only 0.8% *Salmonella* and 1.5% *Shigella* which were highly susceptible to the antibiotics. Our findings regarding the absence of Acid Fast Bacilli on currency notes are contrary to a study conducted in Tanzania but supported by another study from Pakistan.^8^,^14^

None of the *Streptococci* in our research found to be resistant to vancomycin and linezolid. More than 50% of *Staphylococci* and all of the *Streptococci* were resistant to penicillin and ampicillin. These results are in accordance with a study conducted in Bengal, however contrary to this study, none of our Gram-negative bacteria found to be resistant to amikacin, cefoperazone-sulbactam and piperacillin-tazobactam.^5^ In a survey conducted in Saudi Arabia, methicillin-resistant *Staphylococcus aureus* (MRSA), vancomycin-resistant enterococci (VRE) and extended-spectrum beta-lactamase (ESBL) producing bacteria were isolated from Saudi currency notes.^7^ In our study, we detected 33.3% of the methicillin-resistant *Staphylococci* and none of the isolated strain found to be ESBL producing. We observed that our soiled and lower denomination currency notes of rupees 10 were more contaminated than other currency notes which have also been seen in another study.^14^

The currency notes circulating in hospital and community are contaminated with highly pathogenic and some multi-drug resistant bacteria which serve as a vehicle of infectious diseases in community as well as in the hospital environment. Government can help to reduce the burden of infectious diseases by launching the plastic polymer currency notes.^20^

### Limitations

The denominations of higher currency notes could not done due to the limited funds.

## CONCLUSION

The nosocomial contamination of circulating currency notes can push the hospitalized patient into a vicious cycle. We emphasize on the hand washing strategy and use of hand sanitizer before handling the food and when visiting or handling a patient. We must provide the awareness to the people to avoid the use of saliva in counting the currency. The decontamination of currency notes by ultraviolet light or formalin vapours in the banks and markets could help to reduce the transmission of the infectious pathogens.

### Authors’ Contribution

**HE:** Drafted the paper and reviewed the manuscript.

**AJ:** Conceived the idea and data collection.

**MZ:** Designed the study, performed data analysis and writing.
